# A multi-site clinical field study to evaluate the effectiveness of manual cleaning for flexible gastrointestinal endoscopes

**DOI:** 10.1186/2047-2994-4-S1-P54

**Published:** 2015-06-16

**Authors:** M Bommarito, G Thornhill, D Morse, H Reuter

**Affiliations:** 1 Infection Prevention Division, 3M, St Paul, MN, USA; 2 Infection Prevention Division, 3M, Neuss, Germany

## Introduction

An ATP bioluminesce assay (ATP Water Test), measuring organic bioburden including microbes, was used to monitor the cleanliness of flexible GI endoscopes after the manual cleaning step of the decontamination and disinfection process, in seven US hospitals.

## Objectives

To characterize the effectiveness of the manual cleaning step during reprocessing of flexible endoscopes, from site to site as well as by type of endoscope.

## Methods

Duodenoscopes, gastroscopes, and colonoscopes were tested using an ATP Water Test. The method entailed collecting and testing a sterile water sample flushed through the suction/biopsy lumen of the endoscope after completion of manual cleaning. The amount of ATP, in relative light units (RLUs), was measured with a hand-held luminometer. Cleaning failure rates based on a comparison to a published cleanliness pass-fail criterion [1,2], were determined. A total of 398 endoscopes were tested.

## Results

The level of ATP contamination post manual cleaning was found to be statistically different by scope type (p<0.0005), as well as from site to site for each scope type (all p-values < 0.05). Duodenoscopes were found to have the highest mean value of ATP contamination (142 RLUs), followed by gastroscopes (83 RLUs), and finally colonoscopes (29 RLUs). The standard deviation of the mean was also largest for duodenoscopes and gastroscopes. Using the 200 RLUs value proposed in the literature^1,2^ as a pass-fail cleanliness criterion after manual cleaning, the observed failure rates were found to be highest for duodenoscopes (31%, 15/48) and gastroscopes (22%, 38/168) and lowest for colonoscopes (3%, 6/182).

## Conclusion

398 endoscopes, for seven different US hospitals were measured for ATP contamination after manual cleaning. Considerably higher levels of ATP contamination were found in duodenoscopes and gastroscopes as compared to colonoscopes, leading to a significant number of cleaning failures. Given the importance of the manual cleaning step to ultimately achieve proper high level disinfection, these results suggest that protocols and methods used to manually clean upper GI endoscopes may not be adequate.

## Disclosure of interest

M. Bommarito Employee of: 3M, G. Thornhill Employee of: 3M, D. Morse Employee of: 3M, H. Reuter Employee of: 3M.
